# City Scale Modeling of Ultrafine Particles in Urban Areas with Special Focus on Passenger Ferryboat Emission Impact

**DOI:** 10.3390/toxics10010003

**Published:** 2021-12-21

**Authors:** Marvin Lauenburg, Matthias Karl, Volker Matthias, Markus Quante, Martin Otto Paul Ramacher

**Affiliations:** 1 Institute of Coastal Environmental Chemistry, Helmholtz Zentrum Hereon, 21502 Geesthacht, Germany; marvin.lauenburg@hereon.de (M.L.); volker.matthias@hereon.de (V.M.); markus.quante@hereon.de (M.Q.); martin.ramacher@hereon.de (M.O.P.R.); 2 Faculty of Sustainability, Leuphana University Lüneburg, 21335 Lüneburg, Germany

**Keywords:** ultrafine particles, urban air quality, in-land ferryboat emissions, chemistry transport model, particle number size distribution, city scale modeling

## Abstract

Air pollution by aerosol particles is mainly monitored as mass concentrations of particulate matter, such as PM_10_ and PM_2.5_. However, mass-based measurements are hardly representative for ultrafine particles (UFP), which can only be monitored adequately by particle number (PN) concentrations and are considered particularly harmful to human health. This study examines the dispersion of UFP in Hamburg city center and, in particular, the impact of passenger ferryboats by modeling PN concentrations and compares concentrations to measured values. To this end, emissions inventories and emission size spectra for different emission sectors influencing concentrations in the city center were created, explicitly considering passenger ferryboat traffic as an additional emission source. The city-scale chemical transport model EPISODE-CityChem is applied for the first time to simulate PN concentrations and additionally, observations of total particle number counts are taken at four different sampling sites in the city. Modeled UFP concentrations are in the range of 1.5–3 × 10^4^ cm^−3^ at ferryboat piers and at the road traffic locations with particle sizes predominantly below 50 nm. Urban background concentrations are at 0.4–1.2 × 10^4^ cm^−3^ with a predominant particle size in the range 50–100 nm. Ferryboat traffic is a significant source of emissions near the shore along the regular ferry routes. Modeled concentrations show slight differences to measured data, but the model is capable of reproducing the observed spatial variation of UFP concentrations. UFP show strong variations in both space and time, with day-to-day variations mainly controlled by differences in air temperature, wind speed and wind direction. Further model simulations should focus on longer periods of time to better understand the influence of meteorological conditions on UFP dynamics.

## 1. Introduction

Air pollution represents the largest environment-related health risk in Europe, with $4.0\times 10^{5}$ premature deaths annually [1]. Aerosol particles are considered the air pollutant most relevant to health [2,3]. In Germany, particulate matter concentrations have been constantly monitored since 1995 by measuring the particulate mass per cubic meter of outdoor air, and exceedances are recorded in accordance with EU-wide legislation. The definition of PM_2.5_ (particles with aerodynamic diameter (DM) < 2.5 μm) already aims to separately consider smaller particles with both potentially greater health risks and longer transport distance as well as residence time [4,5] than the larger particles included in PM_10_ (particles with DM < 10 μm). Still, ultrafine particles (UFP; particles with mobility diameter less than 100 nm) are not adequately represented in existing mass-based particle monitoring [6,7,8]. In urban areas, the ultrafine spectrum comprises 80–90% of the particle number concentration [9,10], but only a small fraction of the total particle mass. Neither PM_10_ nor PM_2.5_ concentrations exhibit a consistently significant correlation with UFP concentrations, implying that a low mass concentration reading does not preclude a high UFP number concentration [11,12]. UFP number concentrations are much more sensitive to recently discharged emissions, making them a more pertinent emission indicator [12]. As only particle number concentrations cover UFP, there is an increasing necessity for monitoring of particle number (PN) emissions in addition to control of mass concentrations.

Ultrafine particles consist predominantly of organic compounds, with a varying proportion of nitrogen and sulfur compounds and elemental carbon [13,14]. Their small size is particularly critical as they are able to deeply penetrate into the lungs, enter the bloodstream and the cell organelles of humans and, thus, can trigger health problems in several human organs [4,15,16]. Their relatively large surface area facilitates the absorption of toxic organic substances [7]. The study of individual aerosol particles shows that a significant portion of the potential toxicity of aerosol particles relates to ultrafine particles, as they can be the cause of adverse health effects such as asthma, oxidative stress, and atherosclerotic lesions [17,18]. UFPs are therefore considered to be particularly hazardous to human health.

Shipping is one of the major sources of air pollution [19]. In coastal European cities, shipping emissions contribute significantly to PM_2.5_ emissions, e.g., 14% in Sevilla (Spain) and 20% in Genova [20,21]. Regarding UFP concentrations, shipping emerges as a major source in coastal cities, although the number of studies dealing with ship emission impact on UFP is currently much smaller than for particulate matter [22]. Number concentrations are assumed to be a better metric for monitoring ship emission impacts [23] because ship plumes can be better discriminated from the background pollution on the basis of particle numbers. González et al. (2011) [22] found that in coastal cities, up to 70% of UFP concentrations can be linked to in-land transport of ship plumes, reaching concentrations within 3.5–5 $\times 10^{4}$ cm^−3^ (particles per cubic centimeter). Other studies also report increased UFP concentrations due to ship emissions in maritime European cities [24,25].

The vast majority of freshly emitted ship exhaust particles lie in the ultrafine mode [24]. As in-land passenger ferryboats operate close to the shore and at high frequency, they might be of particular importance for UFP concentrations. Available studies report increased UFP concentrations in residential areas and workplaces due to ferryboat traffic [26,27]. Observations of in-land passenger ferryboat traffic in Lisbon showed UFP concentrations within $2–7\times 10^{4}$ cm^−3^ close to piers during the presence of ferries, corresponding to an increase by 25–197% compared to periods with no ferry arrivals/departures [27]. Furthermore, particle number emission factors (EF_N_) exist for different types and sizes of passenger ferries, but mostly for HFO engines or large diesel engines. EF_N_ are at $5\times 10^{15}–1\times 10^{17}$ cm^−3^ [25,28,29]. Nevertheless, research on emission impact, especially of in-land ferryboat traffic, is lacking.

Concentrations of ultrafine particles are influenced by diverse external factors, which makes an impact assessment of one sector in relation to others a challenging task. Often, studies focus on the influence of specific local sources such as roads, industrial facilities, or airports [30,31,32,33]. These studies hardly provide the information needed to identify spatial patterns of the impacts from a specific local pollutant source [34]. Other studies focusing on UFP concentrations from a regional background are showing that different size distributions and higher concentration fluctuations may occur depending on the region, but are usually not addressing specific sources [35,36]. The identification of sources of UFP pollution is further complicated by new particle formation (NPF) events in the urban atmosphere that can have a substantial influence on PN concentrations. The occurrence of NPF events depends on the condensation sink of pre-existing particles, solar radiation, and the availability of precursor gases [10,37,38].

This study aims to (1) simulate area-wide concentrations and identify sources of measured concentrations in order to understand UFP dynamics, and (2) identify concentration maxima and hotspots of UFP pollution. Therefore, the city-scale chemical transport model (CTM) EPISODE-CityChem [39,40] is applied to the city of Hamburg. The application of EPISODE-CityChem allows the impact of passenger ferryboat UFP emissions to be assessed in relation to other sectors. Since passenger ferryboats operate with high frequency close to the shore and in proximity to the city center, they might represent a major UFP contributor to the exposure in populated areas. Furthermore, there are potential measures to mitigate the particle emissions from public transport, e.g., through replacement with electro-ferries. To perform the computer simulations, all relevant emission sectors need to be identified and included in the model as well as the meteorological conditions. Due to the complexity of UFP dynamics and the lack of comparable results of modeled concentrations, this study includes a comparison against PN concentration measurements taken at four different sampling sites within the modeled area.

## 2. Materials and Methods

**Figure A1:**
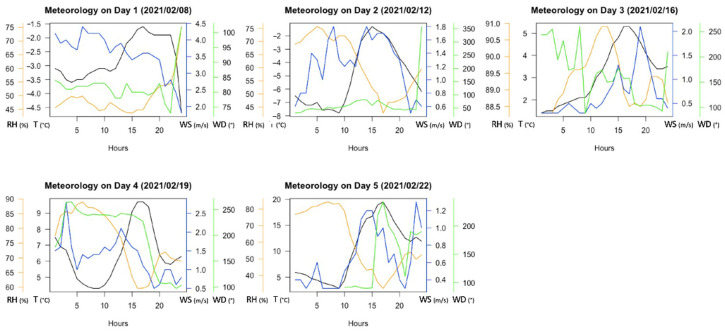
Variation of meteorological conditions on the selected days of this study. Plots for each day show hourly data of wind speed (blue lines), wind direction (green lines), temperature (black lines), and relative humidity (orange lines). Meteorological data from the ground weather station Finkenwerder West.

In this study, the city-scale chemical transport model EPISODE-CityChem [39,40] is applied to model UFP concentrations in Hamburg city center on five days in February 2021. The first objective has been to cover a wide range of meteorological conditions in order to relate changes in meteorology to changes in UFP concentrations. Only precipitation-free weekdays that were characterized by different ambient temperatures, wind speeds and wind directions have been selected in order to obtain a comprehensive picture of conditions with potentially high concentrations. A second objective was to find suitable conditions for the determination of ferryboat impacts. To this end, an upper limit of the daily mean wind speed of 2 m s^−1^ was introduced for selecting days that were suitable for measurements at the ferryboat pier. The investigated five days are distributed over a period of two weeks. Figure A1 shows the hourly variation of the meteorological conditions on the five selected days.

### 2.1. Study Area

The investigated region has an area of 900 km^2^, which covers most of the city of Hamburg, Germany. Hamburg is the second largest city in Germany with a high traffic volume, approx. 1.89 million inhabitants, an international airport and one of the largest ports worldwide; thus, it covers many potential emission sources of UFP. Regular ferryboat traffic is an essential element of the public transport along the city shore. The average annual wind speed of 5−6 m s^−1^ is relatively high compared to the rest of Germany [41]. The annual precipitation is also comparatively high at 668 L/m^2^ [42].

In Hamburg, PM_2.5_ and PM_10_ are continuously monitored by the administrative air monitoring network [43]. Daily mean values for PM_10_ have been largely below the value of 25 μg m^−3^ [44] over the last few decades. Nevertheless, recent studies report relatively bad air quality in Hamburg in a nationwide comparison of annual average concentration (rank 30/51) [45]. At the European level, Hamburg is ranked 125/323 [46]. The highest concentrations are registered at traffic measuring stations and in the city center. For Hamburg, modeled PM_2.5_ concentrations showed a 20−30% share of ship emissions close to the shore [47]. Nevertheless, the only long-term, ongoing measurement series of PN concentrations, spanning several years, in the vicinity of Hamburg is collected at a sampling site located 20 km west of the city center in the town of Wedel on the Elbe River, which only provides information on concentration at one location in Hamburg’s outskirts.

The ferryboats in Hamburg are part of the public transportation system and provide a quick and comfortable alternative to other transportation facilities to move along and across the Elbe River. Especially for dock workers and residents living south of the Elbe River, the ferries are an important connection to the city. Moreover, the ferry route 62, which goes mainly along the northern shore, is a popular route for leisure activities and tourism, reporting the highest number of passengers (HADAG). It is also the longest one, with 12 km on each way. The annual number of passengers on all routes is 9.5 million. The frequency of trips varies among the routes, seasons and days of the week. At daytime, most routes have a 15 or 20 min frequency.

### 2.2. Emission Size Spectra

**Figure 1:**
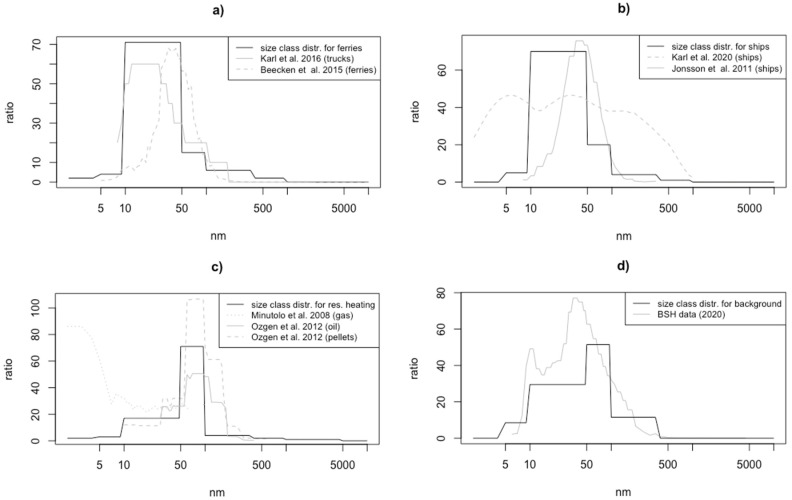
Emission size spectra of different emission sectors (black): ferryboats and road traffic (**a**), ocean-going ships (**b**), residential heating (**c**), and background concentration (BSH station Wedel) (**d**) based on the respective size distributions reported by other studies, provided as grey lines. Solid black lines show the representation of the size distribution in the EPISODE-CityChem model.

The basis for the model is provided by the emission inventories for the various sectors of UFP emissions. Four sectors are considered: ferryboat traffic, ocean-going shipping, road transport and residential heating as these are the sectors that are considered to be most relevant in the city center [48,49]. The calculated annual PN emission totals of the four sectors in the city of Hamburg are $4.1\times 10^{24}$ year^−1^ for road transport, $2.9\times 10^{24}$ year^−1^ for oceangoing shipping, $1.0\times 10^{24}$ year^−1^ for residential heating, and $0.2\times 10^{24}$ year^−1^ for ferryboat traffic. Emission source estimates for PM in the Air Quality Plan of Hamburg cover the mentioned sectors but also include the industrial sector [50]. This study considered the influence of industry emissions indirectly as part of the background concentration (Section 2.2.5). For each sector, the total annual PN concentrations and the emission size spectra of the particles were determined. The size spectra and the size distributions they are based on are illustrated in Figure 1.

#### 2.2.1. Ferryboat Traffic

In total, the ferryboat fleet comprises 25 vessels operating regularly on 7 routes. Each vessel has two 331 kW diesel engines plus 2 assistant engines. Of these, 60% of the fleet is equipped with a selective catalytic reduction (SCR) unit; one is diesel-electric. The particle number emission factor (EF_N_) of ferryboats was adjusted for high-speed/diesel propulsion [51]. EF_N_ for marine diesel engines used on passenger ships are typically 2 (±0.5) $\times 10^{16}$ kg fuel^−1^ with an average peak in PN size distribution between 35 and 54 nm, while the non-volatile fraction peaks is at a smaller size around 10 nm [28,52]. However, these values were measured at large passenger ferries and cruise ships while the considered in-land ferryboats have smaller engines. To the best of our knowledge, the only published study on passenger ferries in a similar size range is from [27], which unfortunately does not include PN size distribution or emission factors for these ferries. Therefore, EF_N_ and size distribution of diesel truck engines were considered. EF_N_ for diesel trucks are in a similar range as the presented EF_N_ for passenger ferries [53], but emission size spectra from diesel truck engines slightly differ from ship diesel engines [25,54]. Nevertheless, it was assumed here that the size distribution of emissions from small ferryboats resembles more the size spectrum of diesel truck engines due to similar engine power. For simplicity, the road traffic emission size spectrum is also used for ferryboats.

#### 2.2.2. Shipping

In port cities, commercial freight and passenger transport by shipping can contribute significantly to PN concentrations in the atmosphere. Ocean-going ships are mostly fueled with heavy fuel oil (HFO), which contributes relatively strongly to particle emissions [29,55]. PN emissions from ships are mostly dominated by the ultrafine spectrum. The EF_N_ for burned HFO ranges from $5\times 10^{15}–5\times 10^{17}$ (L fuel)^−1^ [28,29,56]. The size distribution is characterized by the nucleation mode and the accumulation mode [26]. The average particle diameter of the emissions is in the size range below 50 nm, constituted mainly by particles formed via nucleation of sulfuric acid [24]. The emission size spectra of particles for shipping activities are adopted from [28].

#### 2.2.3. Road Traffic

The most important anthropogenic source of UFP in cities is the transport sector; in European cities, more than half of the particle number emissions can usually be attributed to traffic [48,49]. EF_N_ for light-duty vehicles are at $4\times 10^{14}$ (kg fuel)^−1^ [57]. UFP concentrations related to road traffic typically show a bimodal distribution [58,59]: a first mode in the size range of 13–37 nm formed through gas-to-particle conversion, especially containing sulfates [60], and a second mode in the size range of 64–100 nm, containing, among others, primary particles (mainly soot) as well as droplets of condensable substances from the lubricant oils [59]. However, due to the chemical and physical processes of ultrafine particles, this subdivision is highly variable in size distribution and the boundaries are fluid [61]. The size distribution of road traffic emissions is based on the studies of Karl et al. (2016) [54].

#### 2.2.4. Residential Heating

On average, in Europe, approx. 13% of the number concentrations come from domestic fuel emissions [49]. These depend on the fuels used and their emission factors. In Germany, solid fuels, heating oil and natural gas are the main contributors to particulate emissions [44]. Solid fuels generate the highest emissions per unit of fuel consumed, with approximately $1.5\times 10^{14}$ (L fuel)^−1^, followed by heating oil with approximately $1.5\times 10^{13}$ (L fuel)^−1^ and natural gas with $1\times 10^{11}$ (L fuel)^−1^, whereby the particles from gas combustion are distributed over a particularly small size spectrum [62]. The size distributions are taken from Minutolo et al., 2008 [62] and Ozgen et al., 2012 [63]. Emission spectra of solid fuels and heating oil show a peak at 90 nm emissions; for natural gas, it is significantly smaller at 5 nm. To create a general emission spectrum for this sector, emission spectra of the different heating types were combined according to their share on total emissions. The German federal environmental agency (UBA) provides information on accommodation types. For each accommodation type, an individual demand of fuel type is assumed. Based on the share on demanded energy of each fuel type and its emission factor, the emission share of each fuel type is calculated and weighted in the emission size spectrum accordingly.

#### 2.2.5. Other Emission Sources

Industry and aviation, as two additional sectors of potential importance, have not been considered for three reasons. First, both sectors do not affect the city center directly, as major industry sites and the Hamburg airport are outside the city center. Second, providing annual UFP emissions and respective size spectra for air traffic is challenging because information on jet engines, the wide range of their operation modes and the physical and chemical properties of these particles is poorly documented [64]. Third, PN emissions from the industrial sector do not have a significant impact on the inner-city concentrations. A recent air quality study [47] reported that energy production and industrial combustion processes together are responsible for less than 1% of the total annual PM_2.5_ emissions in Hamburg. Particle emissions of municipal waste incineration and gas-fired plants are low compared to particle emissions of coal-fired plants [65]. In 2021, only one coal-fired power plant located in the eastern part of Hamburg was operative. Model results of PN in a study for several European cities, considering emissions from airports and industrial plants by [66] estimated only 0.2% industrial emission share in the city of Oslo. For these reasons, a constant background concentration of UFP is assumed that is based on measurements in peripheral Hamburg.

The size spectra of each sector consist of eight size classes, among which the model distinguishes. For each of the eight size classes, the associated share of the total PN is given. Ultrafine particles are resolved with the first four size classes. The size classes as well as the distribution of the different sectors are shown in Table 1.

### 2.3. Model Description

The city-scale chemical transport model EPISODE-CityChem combines a 3D Eulerian grid model for area concentrations with a small-scale Gaussian dispersion model for point and line sources [39,67]. Version 1.5 of EPISODE-CityChem was used, which includes the simulation of particle number (PN) concentrations and distributions [68] (available at Zenodo: https://doi.org/10.5281/zenodo.4814060, accessed on 8 September 2021). EPISODE-CityChem predicts hourly concentrations of ultrafine particles and total PN on the 3D Eulerian grid with a horizontal resolution of 1000 m in different vertical layers (first layer with a depth of 17.5 m; top height: 3750 m). At the surface level, a regular receptor grid intercepts PN concentrations at 100 m resolution. Concentrations at the receptor points are calculated as the sum of the background concentration in the corresponding grid cell (derived from the Eulerian grid concentration) and the respective concentration contributions resulting from near-source dispersion of line source and point source emissions (derived from the Gaussian sub-grid models) within a certain influence distance (usually 500 m) around the receptor point.

The output of the model contained the PN concentrations and size distributions for the Hamburg area covering 30 × 30 km^2^ for 5 days in February 2021, with hourly resolution and horizontal resolution of 100 m. An overview of the selected days together with the meteorological conditions is presented in Table 2.

The near-source dispersion of pollutant emissions from line sources (vehicular traffic and ferryboat traffic) is calculated with the steady-state integrated Gaussian plume model HIWAY-2 [69] from US EPA, which can be extended for road traffic by a simplified street canyon model (SSCM). The emitted mass from line sources is integrated into the 3D Eulerian grid model in each simulation time step. The plume rise of the exhaust from ferryboats was expected to be weak and is therefore neglected in this study. For the near-source dispersion of point source emissions, the Gaussian segmented plume model SEGPLU [70] coupled with the Weakwind Open Road Model (WORM) [71] meteorological pre-processor (WMPP) is used. SEGPLU treats the exhaust released from point sources as discrete emissions of finite length plume segments. The plume segments are advected and grow according to the local meteorological conditions; once the expanding plume segments exceeds a predefined size, the plume mass is integrated into the Eulerian grid.

Dry deposition of particles, wet scavenging of particles and coagulation between particles of the same size class are considered. The wet scavenging of particles is parameterized based on the formulation by [72] for the primary aerosol model of the MOCAGE chemistry transport model considering in-cloud scavenging and below-cloud scavenging of particles with different sizes. The wet scavenging implementation is adopted from EPISODE model v10.1 [67]. Dry deposition and coagulation processes were implemented in the Eulerian grid module and the Gaussian modules following the parameterization given in [54]. Coagulation between particles of different size categories is not considered explicitly in EPISODE-CityChem. However, inter-modal coagulation is partly taken into account through the average coagulation coefficient derived from a reference calculation with an aerosol dynamics model that included coagulation between all size sections [54].

### 2.4. Model Input

**Figure A2:**
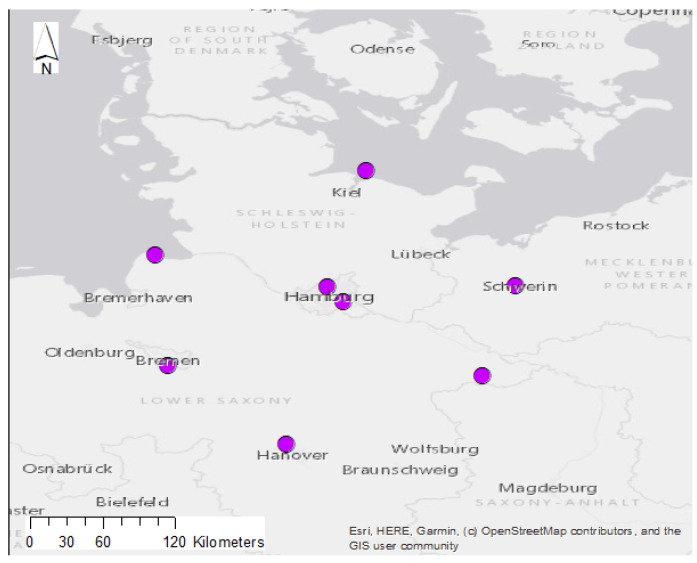
Map showing the locations of the virtual weather stations. The meteorological data extracted from the DWD forecast at the eight virtual weather stations were used as input to the meteorological pre-processor MCWIND.

EPISODE-CityChem reads the meteorological fields to calculate dispersion parameters, vertical profile functions in the surface layer, and eddy diffusivities. Meteorological data on wind speed and direction, temperature, relative humidity, pressure, as well as precipitation for the simulated days were retrieved from the numerical weather prediction data portal of the German Weather Service (DWD) (http://opendata.dwd.de/weather/nwp/icon-d2/, accessed on 8 September 2021) at eight virtual weather stations within a radius of 170 km around the city center of Hamburg (Figure A2).

**Figure A3:**
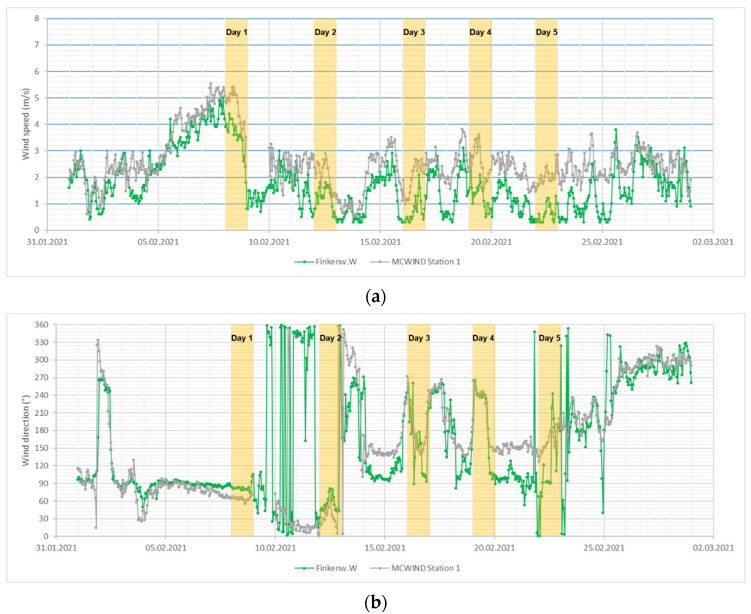
Comparison of (**a**) wind speed (m/s) and (**b**) wind direction (°) used in the model simulation against measurements at weather station Finkenwerder West in Hamburg during February 2021. Missing model data on 9 February 2021. Measurement data obtained from the air quality monitoring network of the city administration (Luftmessnetz Hamburg (HaLM); https://luft.hamburg.de/clp/meteorologie/clp1/, accessed on 8 September 2021). The transparent orange bars highlight the selected days that were investigated in this study.

The built-in meteorological pre-processor, MCWIND, is used to estimate the local wind flow field in the simulation domain based on the input values from eight virtual weather stations, adjusted to the topography of Hamburg, in order to obtain the 3D divergence-free and mass-consistent wind flow field [67]. Other relevant meteorological parameters such as the vertical temperature gradient are also obtained from the MCWIND pre-processor. Accurate wind data are important for simulating the dispersion of pollutants in cities. Figure A3 shows a comparison of the modelled and measured meteorological data at the Finkenwerder West weather station (53.54° N; 9.84° E) in Hamburg. Modelled wind speed and direction agree fairly well with the measurement data, apart from the period of low observed wind speeds (hourly means: 0.4–1.6 m s^−1^; 20–25 February 2021) that tend to be overestimated by the model. One of the selected days of this study, Day 5 (22 February 2021), falls in this period of low observed wind speed. Observed daily mean wind speed on Day 5 was 0.6 ± 0.3 m s^−1^, whereas the predicted daily mean wind speed was 2.0 ± 0.3 m s^−1^.

Model input emissions from the described sectors need to be provided as annual totals. They are then temporally disaggregated using the Urban Emission Conversion Tool (UECT) [67]. Its output consists of hourly emissions of each emission sector individually.

To calculate ferryboat emissions, average driving speed was multiplied by the engine power and the EF_N_ value. By subtracting the calculated driving time between each pier from the time between departures and arrivals according to the schedule, the average retention time at the pier was calculated to be two minutes. It is assumed that, during this time, the assisting engines for maneuvering are active. The sum of emissions from driving and retention time were calculated for all rides on all routes in one year. These were then integrated into the model as line sources along the schedule routes. This way, their location as well as the dilution processes of emission exhaust are taken into account precisely.

Annual ship emissions were calculated using the ship emission model MoSES (Modular Ship Emission Modeling System) [73]. Data from the Automatic Identification System (AIS) were used to determine the fuel consumption based on their movement. Details on the calculation of fuel consumption of ships at berth can be found in [73]. The AIS dataset by the European Maritime Safety Agency (EMSA) for 2015 provided by the Federal Maritime and Hydrographic Agency (BSH) was applied for this study. The cargo turnover at the Port of Hamburg slightly decreased between 2015 and 2021. Assuming a linear relation between emissions and cargo turnover would result in 3% lower ship emissions for 2021 than calculated by MoSES. This deviation is within the error margin of the fuel consumption calculation [73]; hence, the fuel consumption based on AIS data for 2015 was used in this study without correction. To model PN emissions from ships, the fuel consumption of a particular ship was then multiplied by the EF_N_ for the respective ship type. Ferryboat traffic emissions were excluded from the emission inventory for ocean-going ships by identifying ferries based on their Maritime Mobile Service Identity (MMSI) numbers. The MoSES model has already been shown to perform well for particulate matter concentrations [73]. Emissions of ships in the port are integrated into the model as gridded area sources. They are vertically distributed between 1 m and ca. 300 m, according the Expgauss vertical profile [74] for neutral atmospheric stability, assuming a ship stack height of 50 m and a ship building height of 20 m.

Emission data from the transport sector come from the EU Copernicus Atmosphere Monitoring Service (CAMS) emission inventory (CAMS-REG-AP), which contains area-gridded emissions for 2016 of annual NO_X_, PM_10_ and PM_2.5_ emissions, besides other pollutants for different sectors in the gridded nomenclature for reporting (GNFR). To reproduce dilution processes and street canyon effects realistically, road traffic emissions need to be treated as line sources. To transform area-gridded road traffic emissions into line sources, the OpenStreetMap (OSM) database is used. OSM distinguishes between different road types used. All road traffic emissions are distributed to a line segment of the OSM road network, taking into account the total road length and the different road types in the form of weighting factors [75,76]. This way, all area-gridded road traffic emissions are transformed to line sources by top-down distribution, following an approach presented by Ramacher et al., 2021 [75]. Downscaled road traffic emissions are then multiplied with a factor of three in inner-city urban areas to prevent underestimation of NOx traffic emissions in urban core areas through downscaling regional emission inventories [77]. The downscaled and adjusted emissions match well with road traffic emissions based on the inventory of the city of Hamburg 2016. A more detailed description of the downscaling procedure is provided by Ramacher et al., 2020 [47]. Finally, the NO_X_ emissions were used to calculate PN emissions applying a conversion factor of 3 × 10^14^ g^−1^ [78,79].

Residential heating emissions are calculated based on the population tables of the EU Copernicus Urban Atlas 2012 (UA2012) [80,81] and heating type information for Hamburg derived from Census data. The UA2012 dataset contains meta-information on location, number of inhabitants and inhabited area, while the heating type information contains fuel used per household. The annual energy consumption and fuel demand per household can be calculated by multiplying the area by the number of inhabitants with the average energy demand and the calorific value of the fuel, as has been demonstrated for the city of Hamburg by Ramacher and Bieser (2017) [82]. A comparison with the energy demand numbers provided by the city administration indicated that consistent emission values were calculated in the downscaling procedure [50,82]. Due to the overall population increase in Hamburg between 2012 and 2021, we estimate that residential heating emissions might be about 5–6% higher than calculated by the inventory. Offsetting the heating demand with the EF_N_ of the respective fuel gas, heating oil and solid fuels finally results in the emissions per household, which are assigned to the respective grid cell of the model based on their coordinates. All emissions located within a grid cell of the model are summed and distributed to the grid cell as area sources. On a temporal level, the day-to-day variation of residential heating emissions is based on a heating degree-day concept implemented in UECT. This puts in relation the residential heating emissions with outdoor temperature.

Constant background concentrations of particles are used at the lateral and vertical as initial and boundary conditions. The background concentration is derived as average from existing measurement data of PN concentrations gathered by BSH in Wedel (53.57° N; 9.69° E) at the north shore of the Elbe River in the west of Hamburg. The measurement is performed with both a fast mobility particle sizer (FMPS) for particles with diameter from 5 to 300 nm and an optical particle sizer (OPS) for particles with diameter larger than 300 nm. The dataset includes hourly PN concentrations size resolved at 48 channels over a period of half a year (July–December 2020). Measured values were filtered to only include non-riverside wind directions to avoid concentrations affected by ship traffic. Additionally, nucleation events during the measurement period were identified and subsequently excluded. After this procedure, the resulting median PN concentration of 2300 cm^−3^ was used as a constant atmospheric background PN concentration at the boundaries of the model. The median size distribution of the filtered measurement data is displayed in Figure 1d.

**Figure 2:**
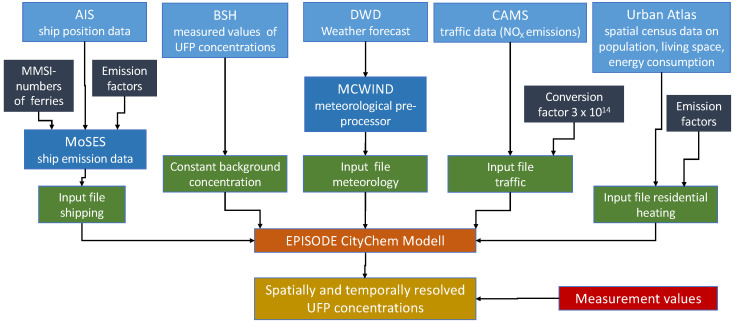
Overview of the model processing chain. Blue: data sources; green: input files; orange/red: output sources; yellow: concentration values.

The overall model setup with the used data sources, model input data, and model output data is shown in Figure 2.

### 2.5. Measurements

In order to compare the model results with real concentrations, measurements were collected at four different sampling sites in Hamburg. As the focus of this study is on modeling UFP, measurements only serve to provide the possibility of evaluating the model performance.

#### 2.5.1. Sampling Equipment

Measurements were made using a P-Trak Ultrafine Particle Counter (Model 8525; TSI^®^, Shoreview, MN, USA), which provides real-time measurements in the size spectrum of 0.02–1 μm in a concentration range of $0–5\times 10^{5}$ cm^−3^. It uses the same technology as a conventional condensation particle counter (CPC). Particles flow through a heated tube, in which the inflowing air is mixed with saturated alcohol vapor. They then enter the condenser, where abrupt cooling causes the alcohol vapor to condense on the particles. This causes the particles to grow into larger and, thus, more easily countable droplets. These drops generate scattered light pulses in the optical measuring cell, which are detected and counted by a photodetector. Further details on the equipment properties can be found at [83]. The device was found to provide reliable information on relative PN concentrations, although it may underestimate concentrations near emission sources [84]. However, the device is a common piece of equipment for (short-term) PN concentration measurements. The device was calibrated by the TSI company shortly before the measurement campaign in Hamburg.

#### 2.5.2. Measurement Sites

**Figure 3:**
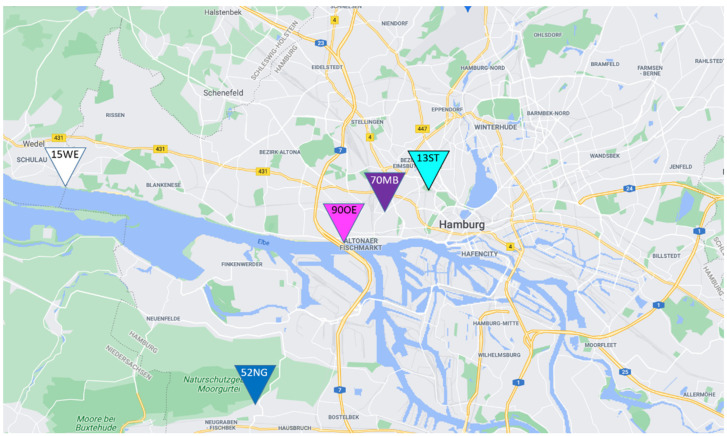
Map of Hamburg with overview of measurement locations. Dark blue: suburban background (Neugraben, 52NG); cyan: urban background (Sternschanze, 13ST); purple: road traffic (Max-Brauer Allee, 70MB); magenta: ferryboat pier (Övelgönne, 90OE); white: background/shipping traffic (location of the measurement data for the background concentration; Wedel, 15WE). The motorway visible on the map right next to the pier is tunneled. The marking in Wedel (15WE) shows the location of constant PN monitoring by BSH that was used for the definition of the constant background concentration.

For the measurements, the particle counter was placed at selected PM measurement stations of the administrative air quality monitoring for Hamburg to ensure the best possible representativeness of sector-specific short-term measurements. An overview of the measurement locations is provided in Figure 3. These include the monitoring stations Sternschanze (13ST), Max-Brauer-Allee (70MB) and Neugraben (52NG). The location of the Övelgönne (90OE) site was defined at our own discretion as there is no administrative monitoring station at a ferryboat pier.

The measuring device was mounted at a height of 2 to 3 m at each measurement site. The measurements took place in the afternoon between 14:00 and 17:00 h local time (UTC + 2). Each measurement site serves to examine an environment exposed to different emission sources, so that the modeled concentrations for the individual sectors can be compared in terms of their consistency. The location at the ferryboat pier was visited on two days as this is the sector that the focus of the study is set on. An overview of the measurement sites and the stations of the administrative network considered in this study is given in Table A1.

The measurements for ferryboat emissions were taken at station 90OE close to the ferry pier at approximately 15 m distance from the landing stages. This pier was chosen because it is solely approached by ferryboats and is located close to residential areas. Since the landing stages for both directions of travel are about 20 m apart, the exhaust fumes from the departures in both travel directions arrive at the site from different wind directions (south or southwest).

The traffic station 70MB is located at a section with buildings 10–15 m high and directly opposite bus stops in both directions of traffic. The distance is only a few meters to the street and about 8 m to the bus stops. Thus, the measurement location is under the influence of a street canyon as well as the exhaust fumes from the regular departure of buses.

The suburban background station 52NG is located north of the district Neugraben and southwest of a nature reserve area, in a non-traveled passageway surrounded by residential buildings. The inner-city urban background station 13ST is located at the southern edge of the Sternschanze city park about 50 m off a dead-end street. The station in Wedel (15WE) is located directly on the shore of the Elbe River, away from local emission sources other than shipping. Although this study focuses on the city center, the stations 52NG and 15WE were selected in order to validate boundary conditions and background concentrations.

## 3. Results

### 3.1. Modeled Spatial Distribution

**Figure 4:**
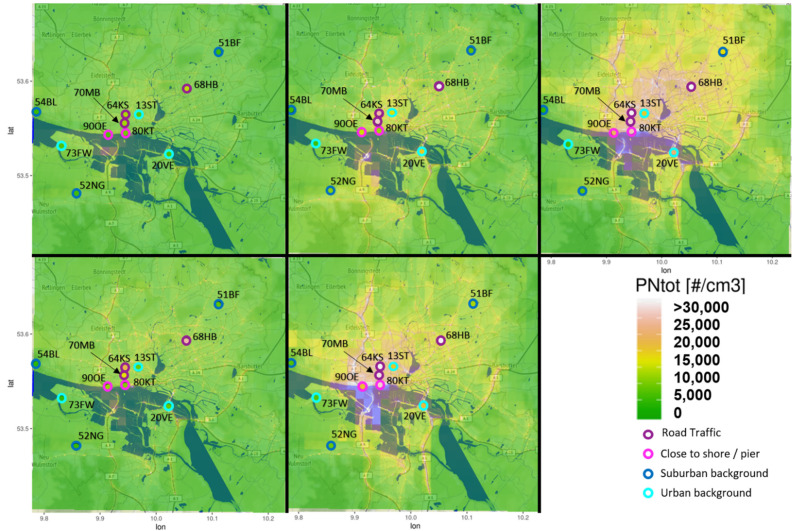
Modelled average particle number concentration (in cm^−3^) in the Hamburg urban area on five days in February; area: 30 × 30 km^2^, resolution: 1 km^2^; blue: Water and harbor area. Circles indicate the locations of stations of the Hamburg air monitoring network. A list of the station acronyms is given in Table A1. The color of the circle outline indicates the station category that the station belongs to (dark blue: suburban; cyan: urban background; purple: road traffic; magenta: close to shore/pier).

The EPISODE-CityChem model was applied twice for each of the five days of measurement in February 2021, first with all emission sources activated and second without ferryboat emissions. For the latter, a model simulation was performed considering all emission sources except ferryboats by excluding ferryboat emissions in the emission input dataset. Figure 4 shows the modeled daily average PN concentrations in Hamburg (30 × 30 km^2^) for each day including all emission sources. Air quality monitoring stations of the city administration (Hamburger Luftmessnetz; HaLM) are marked in Figure 4 as colored dots with their respective code. The modeled PN concentrations reveal large differences between the days, mostly due to high variation in wind speed. In the city center, PN is in the range $1.0–2.5\times 10^{4}$ cm^−3^, in suburban areas $0.3–1.0\times 10^{4}$ cm^−3^. The highest concentrations are found on all days along urban highways and in the port area, where the daily average concentration surpasses $3.0\times 10^{4}$ cm^−3^ at two days. Stations along the shore (90OE, 80KT) show similar high concentrations. In comparison, modeled PN from other urban scale models for comparable cities tend to be slightly lower [85,86]. In accordance with the results presented in this study [66], also reports concentration maxima near major traffic roads in different European cities. Concentration peaks in the port area are also in agreement with results for modeled PN in Oslo and Athens. Like in Oslo, the tunnel entrances show concentration peaks in the modeled spatial distribution. For example, a PN hotspot can be noted at the entrance of the “Elbe Tunnel”, a tunnel that carries a six-lane highway (national motorway A7) beneath the river Elbe.

Differences in the spatial distribution among the five days are notable, e.g., between day 3 and day 5, emissions from residential heating differ (e.g., 51BF, 24FL) probably due to temperature difference. On Day 2 (12 February 2012), despite overall lower concentrations, concentrations in the southwest (e.g., 52NG) are higher due to wind direction from the northeast.

### 3.2. Modeled Particle Size Distribution

**Figure 5:**
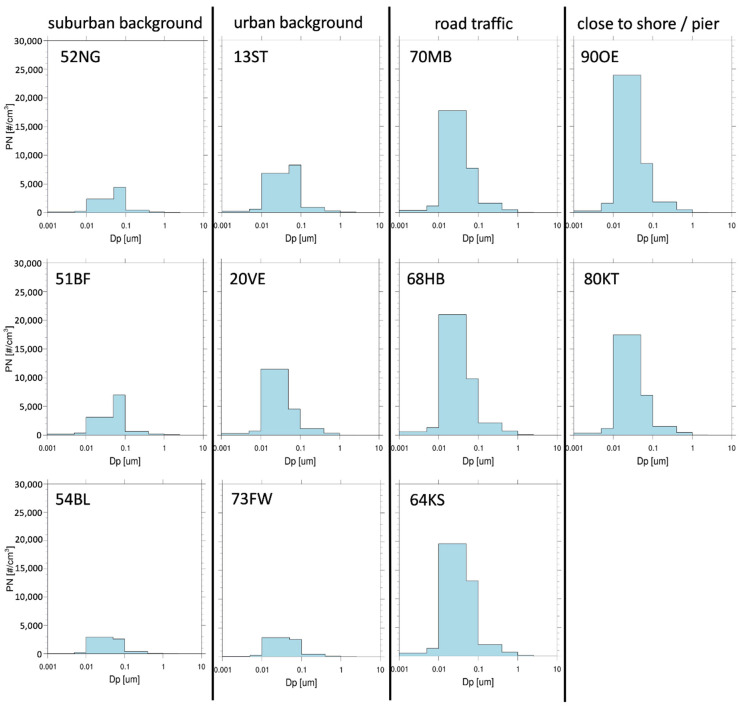
Modeled PN size distributions (on Day 5; 22 February 2021) according to the determined size classes at selected HaLM measurement stations sorted by station category. Top row shows the stations that were selected as PN measurements sites during the campaign. Stations were grouped into four categories: suburban, urban, road traffic and close to shore/pier. A list of the station acronyms is given in Table A1.

The available HaLM stations were grouped into four categories: suburban background, urban background, road traffic and close to shore/pier. Figure 5 shows the modeled size distribution on Day 5 (22 February 2021) for PN at selected HaLM stations, including those chosen for PN measurement. At all stations, PN peaks at size classes 3 or 4, which corresponds to the size range 10–100 nm. Thus, the modeled size distribution allows for a good representation of ultrafine particles in terms of number. The peaks shift according to the category from size class 4 in suburban backgrounds to size class 3 near the road and close to shore/pier stations. Peaks at the urban background locations vary between size class 3 and 4. Accordingly, freshly emitted UFP predominantly have a DM < 50 nm, while suburban background concentrations tend to consist predominantly of particles with DM > 50 nm. Urban background concentrations lie in-between with a balanced ratio of size classes 3 and 4. Stations close to the shore/pier and near road traffic stations show similar size distributions due to the application of the same emission size spectrum for ferryboat emissions and road traffic emissions.

### 3.3. Modeled Diurnal Variation

**Figure 6:**
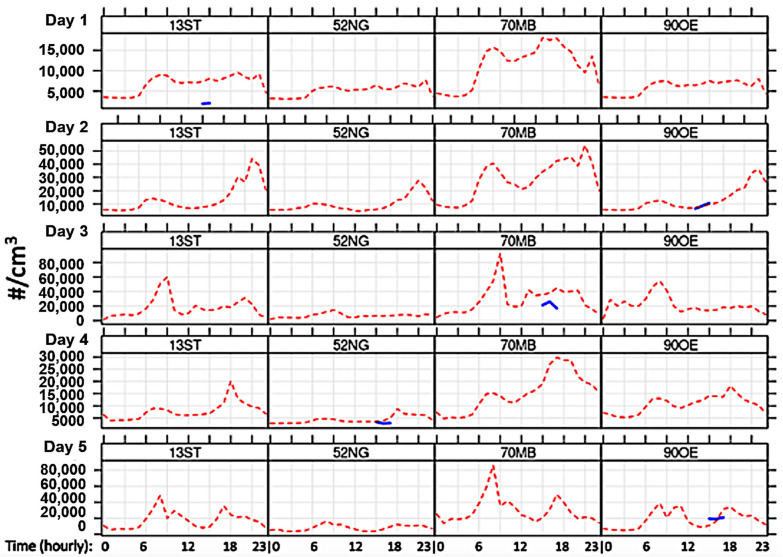
Daily profiles of hourly modeled PN concentrations at the four measurement sites for the five measurement days and the measured PN concentration values at respective daytime. The variation in PN concentrations during the day is mainly controlled by the diurnal variation of emissions and the changes in the meteorological conditions between daytime and nighttime. Red dashed line: model. Blue line segments: measurements. Stations: 13 ST (Sternschanze, urban background); 52 NG (Neugraben; suburban background); 70MB (Max-Brauer Allee, road traffic); 90OE (Övelgönne, close to shore/pier).

Modeled hourly concentrations at the measurement locations are plotted in Figure 6. The dashed red line shows the modeled PN concentration variations over each day at the four measurement stations. Note that the days have different scales for PN. The road traffic station (70MB) shows a bimodal temporal distribution with peaks in the morning and in the evening at rush hour. The ferryboat pier station (90OE) shows most similarities with the inner-city urban background station (13ST). The suburban station (52NG) shows the lowest concentrations and lowest temporal variability. Nevertheless, a general diurnal pattern is recognizable across all stations: the lowest values occur during the night, while maximum values occur in the mornings and evenings. This pattern agrees with findings from other studies [87,88].

As most city-scale models do not consider ultrafine particles and comparison opportunities with PN measurements are scarce, modeled concentrations were compared against the short-term particle measurements to evaluate the model’s accuracy. Measured values are inserted in the diurnal plots of Figure 6 on the respective measurement day and at the corresponding daytime. Average observed concentrations (hourly means) at the ferry pier were $1.05\times 10^{4}$ cm^−3^ (Day 2, 12 February 2021) and 1.96 $\times 10^{4}$ cm^−3^ (Day 5, 22 February 2021) including peaks up to 1.36 $\times 10^{5}$ cm^−3^. With explicit consideration of ferryboat emissions, observed concentrations at the ferryboat pier (90OE) are matched well with measured values. The road traffic station (70 MB) reports the highest observed concentrations with 2.24 $\times 10^{4}$ cm^−3^ on average and peaks up to 2.71 $\times 10^{5}$ cm^−3^. At this station, the model significantly overestimated the measured concentration. However, it should be considered that reduced traffic volume due to measures against the COVID-19 pandemic (for instance, working from home) might have led to lower observed concentrations. The measurement at urban background side (13ST) on Day 1 (8 February 2021) indicates significant overestimation of the measured concentrations by the model too. Low temperatures and high wind velocity from northeastern directions led to extremely low concentrations that the model did not capture accordingly. The extraordinary meteorological conditions prevailing on this day are further discussed in Section 4.3.2. Concentrations measured at the inner-city urban side (13ST) reported 0.2 $\times 10^{4}$ cm^−3^ on average and were therefore lower than observed concentrations at the suburban side (52NG). Measured concentrations from suburban background (52NG) showed an average of 0.3 $\times 10^{4}$ cm^−3^ where model and observation match well.

Further, the goodness of the model was evaluated based on model-observation value pairs of hourly mean PN concentrations (N = 14) using the modstats function of the openair R package [89]. Model predictions are fairly well correlated with observations (Pearson correlation coefficient r = 0.85) and 79% of the predictions are within factor 2 of the measurements (FAC2 = 0.79). The accuracy of model predictions is moderate, as indicated by the root mean square error (RMSE = 8900 cm^−3^) and the mean absolute error (MAE = 5900 cm^−3^), due to the relatively large bias (normalized mean bias NMB = 0.43).

### 3.4. Ultrafine Particles versus Total PN

**Figure 7:**
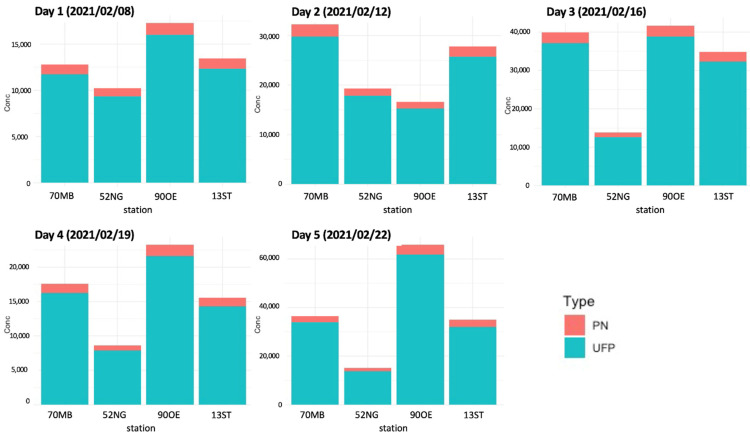
Modeled daily PN concentrations at the four measurement sites for the days 1–5 indicating the UFP share (turquoise) in total particle number counts (red).

For better comparability, concentration results were provided as total PN concentrations. To ensure the representativeness of UFP, Figure 7 shows the share of UFP in the total PN concentration for the five days at the four measurement stations. Its share is found to be almost constant over time and location. On average, the share of modeled UFP was about 91% of total modeled PN. Other studies report a share between 60% and 92% in urban environments [10,12]. A reason for the relatively high share of UFP in this study might be that only a short time period was considered compared to other studies that report long-term observations of particle size distributions including their annual variation [10]. However, for an adequate investigation of the size distribution, a measurement device with size-resolved concentrations would have been necessary.

### 3.5. Contribution of Ferryboats

#### 3.5.1. Share of Ferryboats in the Total Concentration

**Figure 8:**
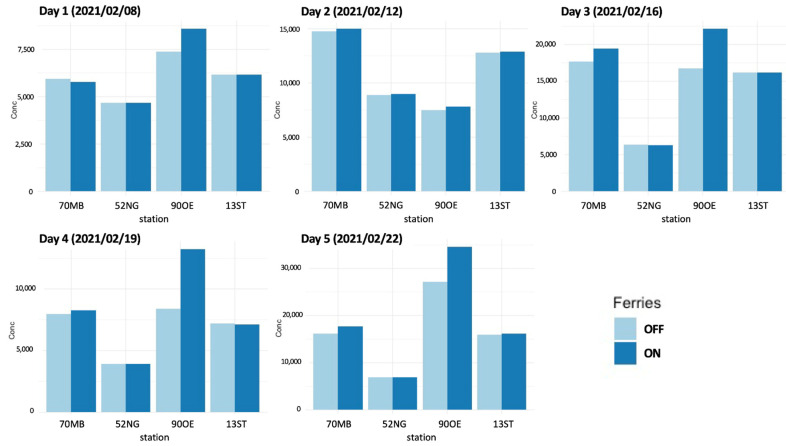
Comparison of modeled daily PN concentrations at the four measurement sites for days 1–5 with ferryboat emissions included (dark blue) and excluded (light blue).

Figure 8 compares modeled PN values with and without consideration of ferryboat emissions at the four measurement stations. At the pier site (90OE), ferryboat emissions account for 13.8%, 6%, 25%, 37.9%, and 23.2% of the total PN concentration on day 1–5, respectively. In absolute concentration numbers, this means an increase in PN of $0.6–0.9\times 10^{4}$ cm^−3^ at the pier under downwind conditions. On Day 1 (8 February 2021) and Day 2 (12 February 2021), wind direction was northeast, which means an upwind position of the measuring site to arriving and departing ferryboats. Here, ferryboat emissions were transported towards the river; hence, its impact was significantly lower at the pier. In comparison, Day 3 (16 February 2021) and Day 5 (22 February 2021) had southern wind directions and Day 4 (19 February 2021) southwestern; all report higher ferryboat emission impact. Wind direction is a key parameter for the impact of ferryboat emissions; directions towards the shore lead to significantly higher concentrations at piers and shores. Figure 8 also reveals a slight increase at the road traffic station (70MB) through ferryboat emissions, except on Day 1. The lower concentrations on Day 1, when ferry emissions are included, might be caused by non-linearity in the coagulation process that removes particles in the model. Days 3 to 5 report higher concentrations in general; therefore, increased particle coagulation took place. The background sites are not influenced by ferryboat emissions. The five-day average contribution of ferryboat emission at the four stations is 21.2% at the ferry pier station (90OE), 4% at the road traffic station (70MB) and <0.1% at the other stations (52NG, 13ST).

#### 3.5.2. Spatial Distribution of Ferryboat Emissions

**Figure 9:**
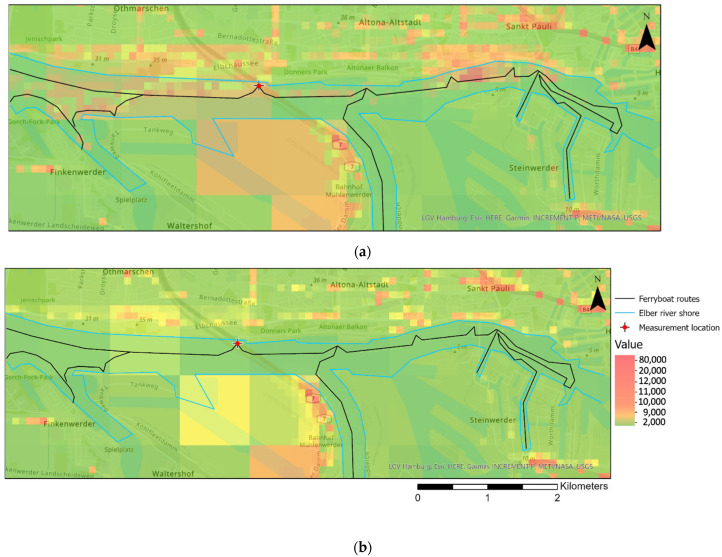
Hourly extract of the PN concentration map for Hamburg on Day 4 (19 February 2021) (**a**) with ferryboat emissions and (**b**) without ferryboat emissions including the Elbe River near the city center as well as the shores. The scale for PN is manipulated for better visualization (see color scale) of the dispersion of ferryboat emissions.

To analyze the spatial distribution of ferryboat emissions, Figure 9 provides an extract of modeled PN concentrations in Hamburg for Day 4 (19 February 2021) including the ferryboat routes (indicated as black lines) and the surrounding shore. On this day, winds arriving from southerly direction with relatively low speed (daily mean wind speed: 1.3 m s^−1^) created conditions for potentially high impacts on the densely populated northern shore. The comparison of PN concentration between modeled PN with (a) and without (b) ferryboat emissions provides insights about their spatial distribution. It should be noted that the scale for the maps in Figure 9 is manipulated in order to achieve enhanced visibility of the spatial emission impact. The densely populated northern shore is clearly affected by the ferryboat emissions. Despite southern wind direction, the port area at the southern shore also shows concentration increases due to the horizontal and vertical diffusion of exhaust particles in the model. Ferryboat emission impacts are mostly notable on the river along the routes and along the northern river shore. This includes most of the ferry piers, including the one where measurements (90OE) were performed. On the northern side of the river, elevated PN concentrations are traceable up to a distance of several hundred meters away from the shore.

PN does not increase equally along the routes. As the route in the east–west direction is by far the most popular route with the highest frequency, it shows the highest increase in PN. In comparison, northeast connections are hardly traceable in the PN concentrations. Nevertheless, there are also striking irregularities in concentration increase within the east–west route. This irregular pattern seems to be related to differences in the upward vertical dispersion along the ferry routes. Hourly mean vertical wind speeds span a range of $0.4–2.0\times 10^{-3}$ m s^−1^ at the extracted hour of the model simulation. The heterogeneity of vertical wind speeds over the Elbe River in the model is due to differences in the elevation of the surrounding terrain and differences in surface roughness over water and land.

## 4. Discussion

### 4.1. Discrepancies between Modeled PN and Measurements

#### 4.1.1. Modeled PN at Traffic Stations

For Hamburg, we found road traffic with a contribution of 50% and oceangoing shipping with a contribution of 35% to be the dominant emission sectors in terms of their total PN emission amount. Highest concentrations were detected at transport locations where the daily average concentration varies in the range of $1.2–3.5\times 10^{4}$ cm^−3^. This aligns with observations in other European cities [32,90,91,92]. Long-term observations at traffic sites in the German cities Leipzig and Dresden report lower values. A six-year measurement (2009−2014) showed an average PN of $1.0\times 10^{4}$ cm^−3^ for February [93], at the lower end of our range. However, the location in Leipzig experiences only half of the traffic volume compared to the observed location in Hamburg, and the location in Dresden does not represent a street canyon. Furthermore, both cities are smaller than Hamburg (population ca. 550,000 in Dresden and 600,000 in Leipzig) and the values include values measured at weekends when particle number is significantly lower [94,95]. The daily PN average near an urban motorway in Berlin (population: ca. 3.77 million) was found to be ~$1.0\times 10^{6}$ cm^−3^ during morning rush hours and ~$4.0\times 10^{4}$ cm^−3^ during weekday afternoons [94]. In our study, the highest modeled concentration for the road traffic site with street canyon effect (70MB) was $8.5\times 10^{4}$ cm^−3^. Other studies recorded concentrations above $1.0\times 10^{6}$ cm^−3^ at peak times near major highways or in street canyons [96,97].

The model results show concentrations mainly influenced by emissions from road traffic and shipping to be dominated by particles with a diameter below 50 nm, which indicates a maximum in the Aitken mode that typically dominates contributions from the road traffic sector [59,98]. This finding indicates that the model treatment of road traffic emissions with Gaussian dispersion and the integrated street canyon effect facilitates a good representation of concentrations along streets and street canyons. The high resolution of 100 m enabled by the integrated small-scale Gaussian dispersion model allows for a good representation of the dynamic PN concentration variation near major emission sources. For a better evaluation of the simulated diurnal cycle of PN concentrations at traffic sites, continuous monitoring with a more advanced particle instrument would be needed.

#### 4.1.2. Modeled PN at Ferryboat Piers

Modeled concentrations at the ferry pier indicate a PN concentration increase of 20 to 40% by ferryboat emissions under downwind conditions. Along the most frequented route of the ferry line network as well as at the piers, the model results show an even higher increase in PN concentrations than found at the measurement site (Figure 8). This indicates ferry routes with high frequency to be an emission source approximately as relevant as highly frequented streets. To our knowledge, the only published study on in-land ferries is for the city of Lisbon (Portugal); it reports concentration increases of a similar range near ferry piers [27]. In Hamburg, the maximum measured concentration at a pier was $4.0\times 10^{4}$ cm^−3^, although the vessels observed in that case have a higher engine power than those in Lisbon.

Interestingly, the model simulations and measurements for Hamburg presented in this study show good agreement at the ferry pier on both days. This indicates both an accurate bottom-up calculation of annual ferryboat emissions and a good representation of ferryboat emissions by treating ferryboat routes as line sources with Gaussian dispersion. However, more measurement locations along the ferry routes as well as longer measurement periods would be necessary to better evaluate the model’s performance on ferryboat emission impact.

#### 4.1.3. Representativeness of Background PN without Temporal Variation

A constant atmospheric background concentration of particles of 2300 cm^−3^ was calculated based on BSH measurements in Wedel (15WE) near Hamburg to account for emissions not considered in the model. Particles in the background air result from natural as well as anthropogenic sources, which are distributed over a large area and cannot be directly assigned to a specific emission source. Measurement stations of the German Ultrafine Aerosol Network (GUAN) [93,94] in suburban background locations show a usual atmospheric background concentration range from 850 to 5000 cm^−3^, depending on how much they are influenced by anthropogenic sources [58,93]. At the stations close to the western border of the model domain, such as Blankenese (54BL), daily averages of $0.3–0.45\times 10^{4}$ cm^−3^ were determined by the model. This is in close agreement with the measured background concentration in Neugraben (52NG) and other studies that have been carried out in comparable areas at the same time of year [88,99]. Regional background PN for Europe presented in [66], modelled with the LOTUS-EUROS chemistry-transport model [100], showed reasonable results for regional background concentration at various locations in Europe, despite existing uncertainties, e.g., parametrization of nucleation processes or secondary aerosol formation from biogenic emissions. These simulations included aerosol process parameterization and showed a substantial contribution of the regional background to total PN in Europe. Modeled monthly mean concentrations at most of the selected regional background stations report of $0.2–0.4\times 10^{4}$ cm^−3^ with little fluctuation [66].

The sites for measuring background concentrations (13ST, 52NG) in the present study have values predominantly at $0.15–0.4\times 10^{4}$ cm^−3^, similar in magnitude to the averaged background concentration at the Wedel site and consistent with other studies of the suburban background [10,48,93]. The measurement in the urban background (13ST), however, is lower than values from the suburban background and is also lower than values presented in other studies that refer to comparable environments [48,101]. This deviation points to limitations of assuming a static background concentration. High fluctuations in the concentration of incoming air masses are not captured in the model. The causes for such an event of high fluctuation that occurred on Day 1 (8 February 2021; when the urban background measurement at 13ST took place) are further discussed in Section 4.2.

The size distribution has the maximum in the range of 50–100 nm and is thus dominated by the accumulation mode. In addition, the diurnal variation was small, confirming a small influence of locally acting sources. Other studies have identified similar characteristics for background areas, inferring sources such as aged emissions from upwind combustion sources, with a unimodal distribution showing a maximum between 80 and 100 nm in diameter [102].

### 4.2. Influence of Meteorology on Modeled PN

#### 4.2.1. Day-to-Day Variations

The difference in the modeled concentrations among the selected days as well as the differences in the two measurements at station 90OE close to the ferry pier show that the number concentrations are highly depending on the meteorological conditions. Wind direction and wind speed are known to be the most important meteorological factors [102,103]. They were found to have the greatest impact on modeled concentrations of this study. Days with higher wind speeds were associated with lower PN concentrations in the model. On Day 1 (8 February 2021), the average wind speed was by far highest (4.4 m s^−1^); on this day, PN was city-wide by 60 to 70% lower than the average of the five days. In contrast, Day 3 (16 February 2021) and Day 5 (22 February 2021) show the highest PN values; during these days, wind speed was relatively low (2 to 4 m s^−1^). The model moderately captures changes in dilution processes by the atmospheric transport of particles due to increased wind speed.

In addition to speed, wind direction, of course, plays a decisive role. Day 2 (12 February 2021) was characterized by a northerly wind; although the total concentration was lower on this day, the values in the south/southwest of the domain were higher than on Day 5. A progression of the pollution plume from the city center in a southwesterly direction can be noticed. Due to wind direction, the location of the port as a major emission source plays a key role for UFP exposure in the city center. During days dominated by southerly/southwesterly wind directions, the exhaust plumes from the port area are carried north towards the city center leading to increased concentrations there (Day 3 and 5).

#### 4.2.2. Clean Air Event on Day 1

**Figure 10:**
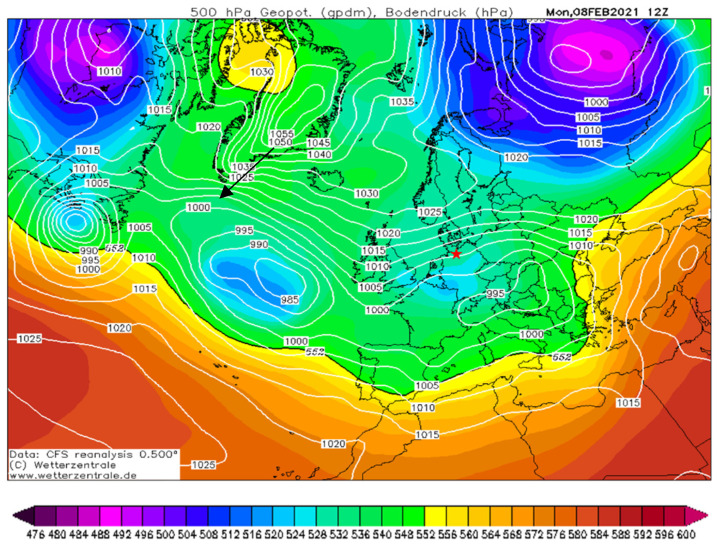
Map of the 500 hPa-geopotential height (in gpdm) and surface pressure (in hPa) for Day 1 (8 February 2021) when an easterly flow of clean polar air led to low PN concentrations in the area of interest. A relatively weak low-pressure system centered over Southeastern Europe and a strong high pressure over Greenland dominated the flow in Northern Europe. (Source: German Weather Central, www.wetterzentrale.de, CFS reanalysis; https://www.wetterzentrale.de/reanalysis.php?jaar=2021&maand=2&dag=8&uur=1200&var=1&map=1&model=cfsr, accessed on 26 November 2021). The red star denotes the area of Hamburg.

The greatest daily variation in modeled overall PN concentration occurred on Day 1 (8 February 2021). Although high wind speed led to low concentrations, the comparison of models and measurements indicates a much stronger model–observation discrepancy in comparison to the other days. Hence, the model was not able to capture the prevailing conditions on this day. For Day 1, the monthly climate status by DWD reports a brisk easterly flow that increasingly steered arctic polar air into the northern half of Germany [104] (see Figure 10). Citywide measurement stations for PM_2.5_ show the lowest values for this day [43]. The extremely clean air from the arctic pole [105] caused an actual decline in concentrations. Even central locations in Hamburg that were located downwind of emission sources show significant changes in concentration and these meteorological conditions with clean air inflow. Under these conditions, model boundary conditions were no longer appropriate for reproducing the real concentrations. This indicates the importance of using the advected background concentration besides inner-city emission sources.

#### 4.2.3. Challenges for Adequate Modeling of Meteorology

The complex emission situation of UFP entails challenges in their modeling. Precipitation, wind velocity, wind direction, and temperature determine how quickly particles are transported or washed out. Higher wind speeds generally lower the concentrations as aerosol particles are transported and diluted more quickly [106,107]. The current study was carried out in late winter during a relatively cold episode. Primary emissions are more significant in winter months, as domestic heating and traffic cause higher emissions. The vertical stability of the atmosphere affects how quickly particles reach the upper layers and are transported away at a correspondingly faster or slower rate. Although the model has a reasonable vertical resolution, situations characterized by a stable stratification in particular may not be represented adequately, which may lead to discrepancies between model results and measurements. In winter, inversions occur more often than in other seasons, resulting in a very stable atmospheric stratification. If this is enhanced by the enrichment of air with water vapor and weak solar radiation, air pollutants are hardly transported vertically and accumulate in the atmospheric layer near the ground [90]. Furthermore, lower ambient temperatures lead to faster saturation of condensable vapors (e.g., from traffic exhaust), resulting in higher traffic-related UFP concentrations in the nucleation mode. For these reasons, concentrations dominated by traffic emissions are found to be higher during winter months [108]. Year-round measurements in other cities have shown that concentrations in winter are up to twice as high as in summer; in the smallest size range below 30 nm in particular, the particle number is larger [109].

### 4.3. Limitations of the Current Methodology Application

#### 4.3.1. Missing Emissions from Airport Activities

Aircrafts emit significant amounts of ultrafine particles. At large airports, $5\times 10^{4}$ cm^−3^ has been measured downwind, and the takeoff and landing of individual aircraft can be clearly traced in the concentration trends [110]. The size spectrum of the particles from aviation is predominantly distributed in the range below 40 nm, often below 20 nm [64,110]. In particular, the spatial extent of aviation emissions has long been underestimated; near airports, significantly elevated concentrations could still be detected downwind for several kilometers [111]. Therefore, emissions from Hamburg airport might be of relevant influence despite the airport location being several kilometers away from the city center.

However, determining accurate emission levels from aircraft is challenging for several reasons. First, there are various technical differences, and second, the physical and chemical properties of UFPs are poorly documented [64]. Therefore, aircraft emissions were not considered in the model of this study. Emission inventories for airports could be an important step towards more accurate PN modeling.

#### 4.3.2. Nucleation Events

Secondary particles related to large-scale NPF events are more likely to occur in summer [87,112] and in high-insolation regions such as southern Europe [113]. NPF events in the urban background air are frequently associated with a prominent nucleation mode with peak diameter below 10 nm [114]. Such nucleation events are not considered in the EPISODE-CityChem model yet, but there are plans to implement the aerosol dynamics code MAFOR in the future development of the model [39].

Source attribution of UFP concentrations is a complex undertaking because of the highly dynamic sequence of chemical and physical processes such as nucleation, condensation, deposition, and coagulation [58,61]. The mentioned occurrence of NPF events is a particular driving factor that can be of high relevance for PN concentrations and that are difficult to cover in model simulations on the urban scale, since they are often a large-scale phenomenon.

## 5. Conclusions

Air pollution is a major health risk in urban living spaces. Although UFP are considered to be of particular harm for human health, air quality monitoring mainly takes place in terms of mass-based concentration units, which are hardly representative for UFP exposure. Modeling particle numbers can help to capture und monitor UFP exposure in cities. Based on modeling and measurements in this study, emissions from regularly operating ferryboats might present a significant contribution to UFP concentrations.

In this study, air pollution by UFP in Hamburg was investigated using the city-scale chemical transport model EPISODE-CityChem to PN. Five days of February 2021, in late wintertime, were selected for modeling and examining short-term measurements in order to take into account different meteorological conditions. Input data for major emissions sources in Hamburg city center as well as the respective emission size spectra were provided based on existing emission data, sector-specific bottom-up calculation, and the shipping emission model MoSES. Input data for meteorology were retrieved from the numerical weather prediction data portal of the German Weather Service.

Modeled concentrations for Hamburg are in a similar range to European cities of comparable size. Hourly average PN appears to be highest in the city center and in the port area; however, there was a great variation between the days. Maximum values of UFP concentration from both model and short-term measurements are found at locations near major highways. Ferryboat emissions represent a dominant emission source near the shore along the routes and especially at ferry piers. The recorded PN concentrations at a ferry pier are in the range of $1.0–3.0\times 10^{4}$ cm^−3^ and, therefore, in a range similar to concentrations near city road traffic. In this context, meteorological parameters such as wind speed and direction as well as temperature represent the major cause of day-to-day variability of UFP concentrations over a wide range. Like road traffic emissions, concentrations dominated by ferryboat emissions mostly consist of particles below 50 nm and, consequently, are hard to capture with mass-based concentrations.

Size-resolved PN measurements using a CPC device would be the accurate next logical step to better observe UFP in urban areas and to supplement the city-scale model. By doing so, two major issues of the presented measurements could be remediated. First, the provided emission size spectra for different emission sectors could be examined by taking size distributed measurements at locations dominated by the respective emission sources, to provide more realistic emission size spectra to the model. Second, potential underestimations of total PN due to device limitations could be excluded. In particular, at road traffic locations with high numbers of small particles, the applied particle sensor shows limitations in accurately counting the numerous nucleation mode particles and this is where model and measurement deviate significantly in this study.

On a methodological level, the city-scale CTM EPISODE-CityChem turned out to be certainly suitable for the realistic representation of the spatial variability of PN concentrations. Deviations to measurements occurred mainly in inner-urban areas and at traffic stations, where observed PN was overestimated, which is due to methodological limitations and differences in the representativeness of the modeled concentrations, i.e., model grid volume versus point location measurement.

## Figures and Tables

**Table 1:** Overview of the 8 size classes and their percentage share in the various emission sectors. Given shares are cumulative over the size distribution.

Area (nm)	Background	Residential	Street/Ferryboat	Ships
<5	0%	2%	2%	0%
<10	8.5%	5%	6%	5%
<50	38%	22%	77%	75%
<100	89.5%	93%	92%	95%
<400	100%	97%	98%	99%
<1000	100%	99%	100%	100%
<5000	100%	100%	100%	100%
<10,000	100%	100%	100%	100%

**Table 2:** List of the selected days of this study together with the meteorological conditions and the PN measurement site. Wind speed, wind direction, temperature, and relative humidity are given as daily mean of the respective day. In brackets are the minimum and maximum hourly values recorded on that day.

Day (Date)	Wind Speed (m/s)	Wind Direction (°)	Temperature (°C)	Rel. Humidity (%)	PN Measurement Station
Day 1 (8 February 2021)	4.4 (0.8–5.2)	83 (73–105)	−2.8 (−5.3–−1.6)	50.5 (43.6–76.3)	Sternschanze (13ST)
Day 2 (12 February 2021)	1.2 (0.5–1.8)	65 (31–81)	−5.1 (−7.8–−1.3)	61.6 (42.7–75.7)	Övelgönne (90OE)
Day 3 (16 February 2021)	0.7 (0.3–2.1)	167 (89–260)	3.2 (1.4–5.3)	89 (83–91)	Max-Brauer-Allee (70MB)
Day 4 (19 February 2021)	1.3 (0.5–2.8)	198 (97–265)	6.4 (4.3–9.7)	75.7 (59.6–89)	Neugraben (52NG)
Day 5 (22 February 2021)	0.6 (0.3–1.3)	182 (91–242)	10.3 (2.8–19.5)	60.8 (32.2–84.3)	Övelgönne (90OE)

## Data Availability

The data presented in this study are available on request from the corresponding author.

## Appendix B. List of the Monitoring Stations Considered in This Study

**Table A1:** List of the monitoring stations of the HaLM network and the measurement sites considered in this study, including information on station category, station location (coordinates in decimal degrees) and a short description of the station. The date of the measurement is given for the stations where measurements of PN concentrations took place.

Station	Code	Category	Coordinates	Properties	Measurement Days
Sternschanze	13ST	Urban background	53.56; 9.96	Residential heating; in a park without influence of direct emission sources	Day 1 (8 February 2021)
Övelgönne	90OE	Close to shore/pier	53.54; 9.91	Ferryboat emissions; located at the Övelgönne pier, passively influenced by urban background	Day 2 (12 February 2021) and Day 5 (22 February 2021)
Max-Brauer Allee	70MB	Road traffic	53.55; 9.94	Road traffic emissions; enclosed by a busy four-lane road, passively influenced by urban background	Day 3 (16 February 2021)
Neugraben	52NG	Suburban background	53.48; 9.85	Background concentration; on the edge of a suburban neighborhood and a nature reserve	Day 4 (19 February 2021)
Wedel BSH	15WE	Suburban background	53.56; 9.71	Bases for boundary conditions, on the edge of the model extent	June–December 2021
Bramfeld	51BF	Suburban background	53.63; 10.11	Within the suburbs in the Northwesterly edge of the city	-
Blankenese	54BL	Suburban background	53.56; 9.78	In a quarter at the left edge of the model domain	-
Finkenwerder	73FW	Urban background	53.53; 9.83	At a central location at the southside of the river	-
Habichtstraße	68HB	Road traffic	53.59; 10.05	At a major traffic lane in a densely populated area in the northwest	-
Altona Elbhang	80KT	Close to shore/pier	53.54; 9.94	Halfway up the slope along the northern shore of the river	-
Kieler Straße	64KS	Road traffic	53.56; 9.94	Next to a four-lane street with street-canyon effect	-
Veddel	20VE	Urban background	53.52; 10.02	In a residential surrounded by mainly surrounded by port area	-
